# circPUM1 Promotes Tumorigenesis and Progression of Ovarian Cancer by Sponging miR-615-5p and miR-6753-5p

**DOI:** 10.1016/j.omtn.2019.09.032

**Published:** 2019-10-23

**Authors:** Xue Guan, Zhi-hong Zong, Yao Liu, Shuo Chen, Li-li Wang, Yang Zhao

**Affiliations:** 1 Department of Gynecology, the First Affiliated Hospital of China Medical University, Shenyang 110001, China; 2 Department of Biochemistry and Molecular Biology, College of Basic Medicine, China Medical University, Shenyang 110013, China

**Keywords:** circRNA, ovarian cancer, exosome, microRNA, tumorigenesis, progression

## Abstract

Circular RNAs (circRNAs) have been reported to participate in the molecular mechanism of human cancers. The *PUM1* gene has been confirmed to be closely related to tumorigenesis and progression of ovarian cancer. In the present study, we explored the function and underlying molecular mechanism of circPUM1 in ovarian cancer. qRT-PCR analysis showed upregulation of circPUM1 in ovarian cancer tissues compared with normal ovaries. Gain- and loss-of-function experiments indicated that circPUM1 increased cell proliferation, migration, and invasion and inhibited cell apoptosis. Intraperitoneal injection of circPUM1-knockout tumor cells in nude mice resulted in a decrease in the metastatic ability of the tumor. Bioinformatics analysis and dual-luciferase reporter assays revealed that circPUM1 upregulated the expression of nuclear factor kappa B (NF-κB) and MMP2 by sponging miR-615-5p and miR-6753-5p. Further studies showed that exosomal circPUM1 acted on peritoneal mesothelial cells and increased tumor metastasis. In conclusion, our study indicates that circPUM1 not only promotes ovarian cancer proliferation, migration and invasion, but also acts on the peritoneum and contributes to metastasis of cancer in the form of cancer-derived exosomes.

## Graphical Abstract

**Figure:**
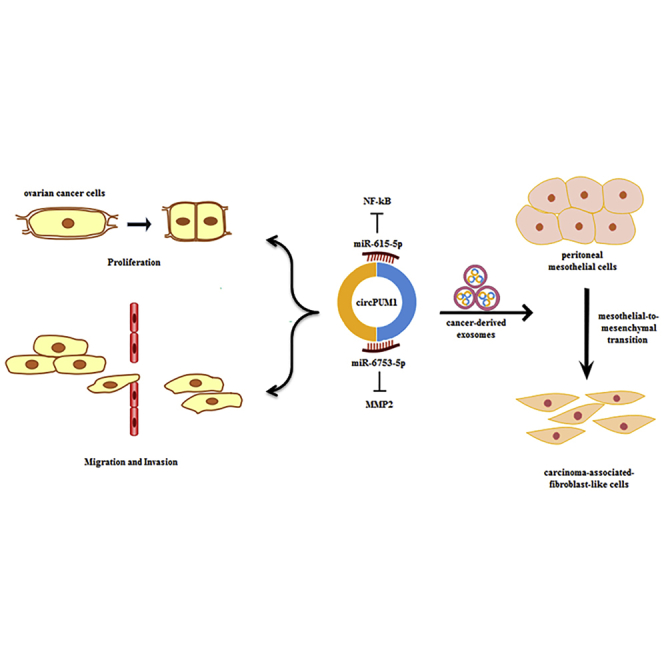

## Introduction

Epithelial ovarian cancer (EOC) is a common gynecologic malignancy. In the United States, for example, it is estimated that approximately 14,080 patients died from ovarian cancer in 2016.^1^ A majority of patients were diagnosed at advanced stages with widespread peritoneal dissemination and metastasis, which largely accounts for the highest mortality rate of this condition among all gynecological malignancies.^2^^,^^3^ Therefore, an improved understanding of the molecular mechanism of the occurrence and development of ovarian cancer is of critical importance.

Increasing numbers of endogenous circular RNAs (circRNAs) have been identified in human cancers.^4^ circRNA is characterized by its unique loop structure without susceptible 5′ or 3′ ends, which leads to its tolerance to exonucleases, as well as greater stability compared to its homologous linear RNA.^5^^,^^6^ Because of its stable structure and variety of microRNA (miRNA)-binding sites, circRNA has been confirmed to participate in the regulation of gene expression, as well as the initiation and development of cancer.^7^^,^^8^

Exosomes are extracellular vesicles ranging from 30 to 100 nm in diameter and containing various functional biomolecules, including proteins, lipids, DNAs, and RNAs.^9^^,^^10^ With the ability to transfer active biological molecules to target cells in distant organs or adjacent cells, exosomes are widely involved in intercellular communication.^11^ Tumor-derived exosomes function as mediators of tumor metastasis by constructing a favorable environment for tumor survival, angiogenesis, and invasion.^12^ Recent studies have shown that circRNAs can be transferred into exosomes.^13^ Li et al.^14^ also reported significant enrichment of circRNAs in exosomes, compared with intracellular levels. Thus, exosomes can amplify the biological effects of circRNAs and mediate their systemic effects. However, the expression profile and biological function of circRNAs, as well as their form in exosomes, remain largely unknown in ovarian cancer.

In this study, we focused on circPUM1, which is derived from exonic back-splicing of the *PUM1* gene, and is the form present in cancer cell-derived extracellular vesicles. The *PUM1* gene is highly expressed in ovarian cancer tissues and closely related with ovarian cancer cell proliferation, migration, and invasion ability.^15^ In this study, we further investigated the function and underlying molecular mechanism of exosomal circPUM1 in tumorigenesis and the progression of ovarian cancer.

## Results

### Correlation of circPUM1 Expression with Epithelial Ovarian Carcinoma

**Figure 1:**
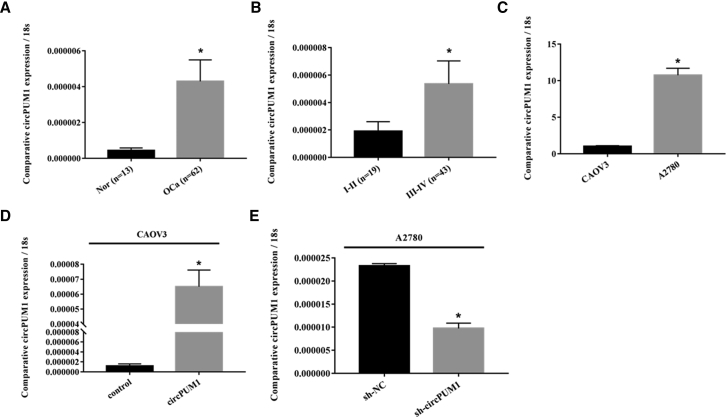
Correlation of circPUM1 Expression with the Development and Progression of Epithelial Ovarian Carcinoma (A and B) circPUM1 expression in ovarian cancer tissue was higher than that in normal tissue (A) and was positively related with FIGO stage (B). circPUM1 expression was higher in the A2780 cell line compared with that in the CAOV3 cell line (C). After pLCDH-circPUM1 plasmid transfection, qRT-PCR revealed that circPUM1 was highly expressed in CAOV3 cells (D). After sh-circPUM1 transfection, circPUM1 was significantly downregulated in A2780 cells (E). *p < 0.05.

qRT-PCR analysis showed that circPUM1 expression was significantly higher in ovarian cancer tissues than in normal ovarian tissues (Figure 1A, p < 0.05, details can be found in Table S1). Moreover, circPUM1 expression was positively related with FIGO stage in ovarian cancer (stage I/II versus stage III/IV, Figure 1B, p < 0.05, details can be found in Table S2).

### circPUM1 Affects Ovarian Cancer Cell Viability, Apoptosis, Migration, and Invasion Ability

**Figure 2:**
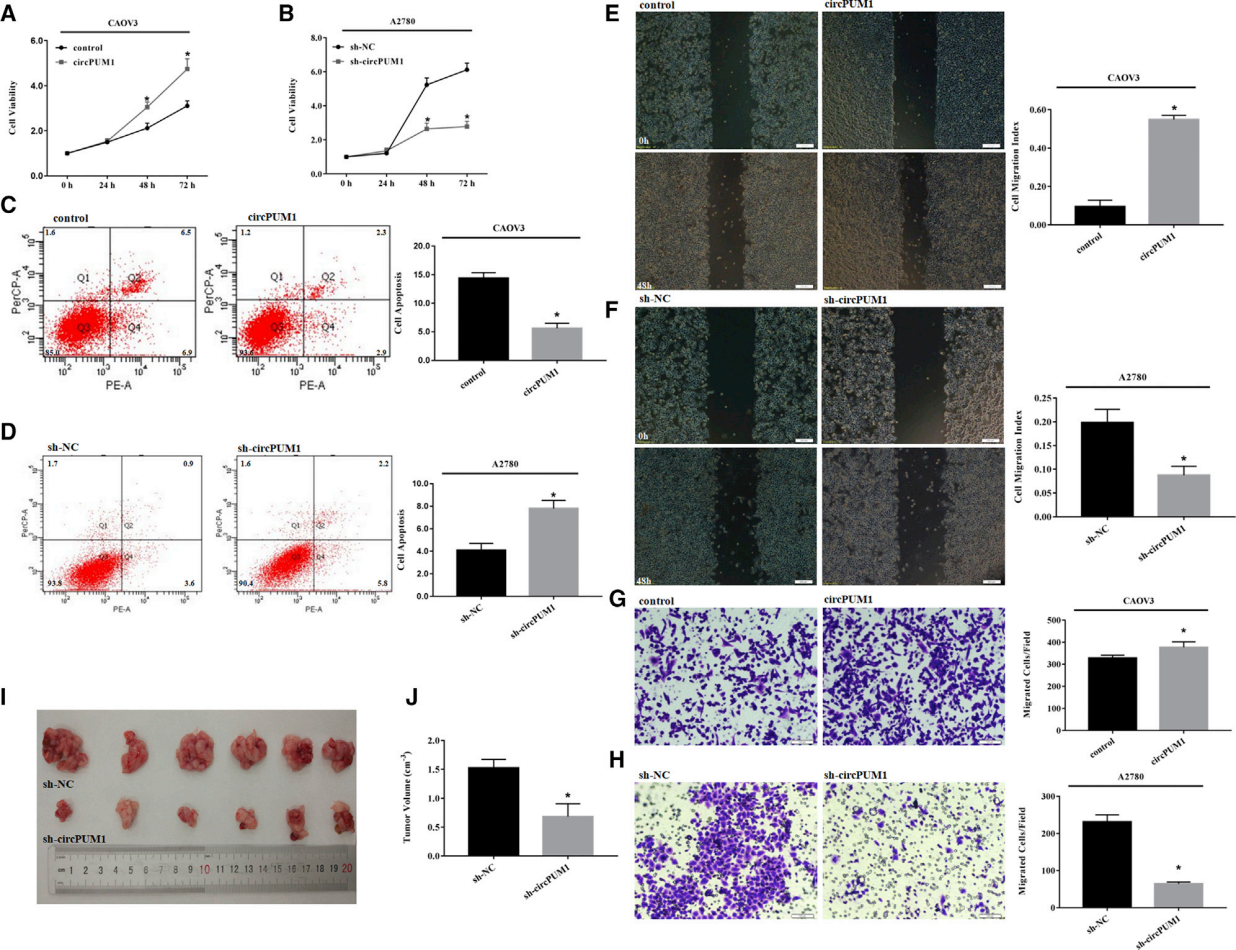
Effects of circPUM1 on Ovarian Cancer Cell Viability, Apoptosis, Migration and Invasion Ability (A, C, E, and G) Compared with the control group, circPUM1 overexpression in the CAOV3 cell line promoted cell growth (A), inhibited cell apoptosis (C), increased cell migration (E) and invasion (G). Downregulation of circPUM1 by shRNA in the A2780 cell line had the opposite results (B, D, F, and H). The results shown are representative of three separate experiments; data are expressed as mean ± SD. Following i.p. injection of female BALB/c nude mice with circPUM1-knockdown A2780 cells, the total number of tumor nodes was lower and metastatic lesions were more limited than those in mice injected with mock transfected cells (I). Following i.p. injection of female BALB/c nude mice with circPUM1-knockdown A2780 cells, the tumor volume was smaller than that in mice injected with mock transfected cells (J). *p < 0.05.

qRT-PCR analysis revealed high expression of circPUM1 in the A2780, while the CAOV3 cell line expressed low levels (Figure 1C, p < 0.05). After pLCDH-circPUM1 plasmid transfection, circPUM1 was highly expressed in CAOV3 cells (Figure 1D, p < 0.05). Similarly, after sh-circPUM1-GFP transfection, circPUM1 was significantly downregulated in A2780 cells (Figure 1E, p < 0.05). Compared with the control group, circPUM1 overexpression promoted CAOV3 cell growth (Figure 2A, p < 0.05), inhibited apoptosis (Figure 2C, p < 0.05), and increased migration (Figure 2E, p < 0.05) and invasion (Figure 2G, p < 0.05). In contrast, the opposite effects were observed following short hairpin RNA (shRNA)-mediated downregulation of circPUM1 in A2780 cells (Figures 2B, 2D, 2F, and 2H, p < 0.05).

### Effects of circPUM1 on *In Vivo* Tumor Growth and Metastasis

Following intraperitoneal (i.p.) injection of female BALB/c nude mice with circPUM1-knockdown A2780 cells, macroscopic observation indicated that the total number of tumor nodes was lower and metastatic lesions were more limited than those in mice injected with mock transfected cells (Figure 2I, p < 0.05). Furthermore, the tumor volume was smaller than that in mice injected with mock transfected cells (Figure 2J, p < 0.05).

### circPUM1 Sponges miR-615-5p and miR-6753-5p

**Figure 3:**
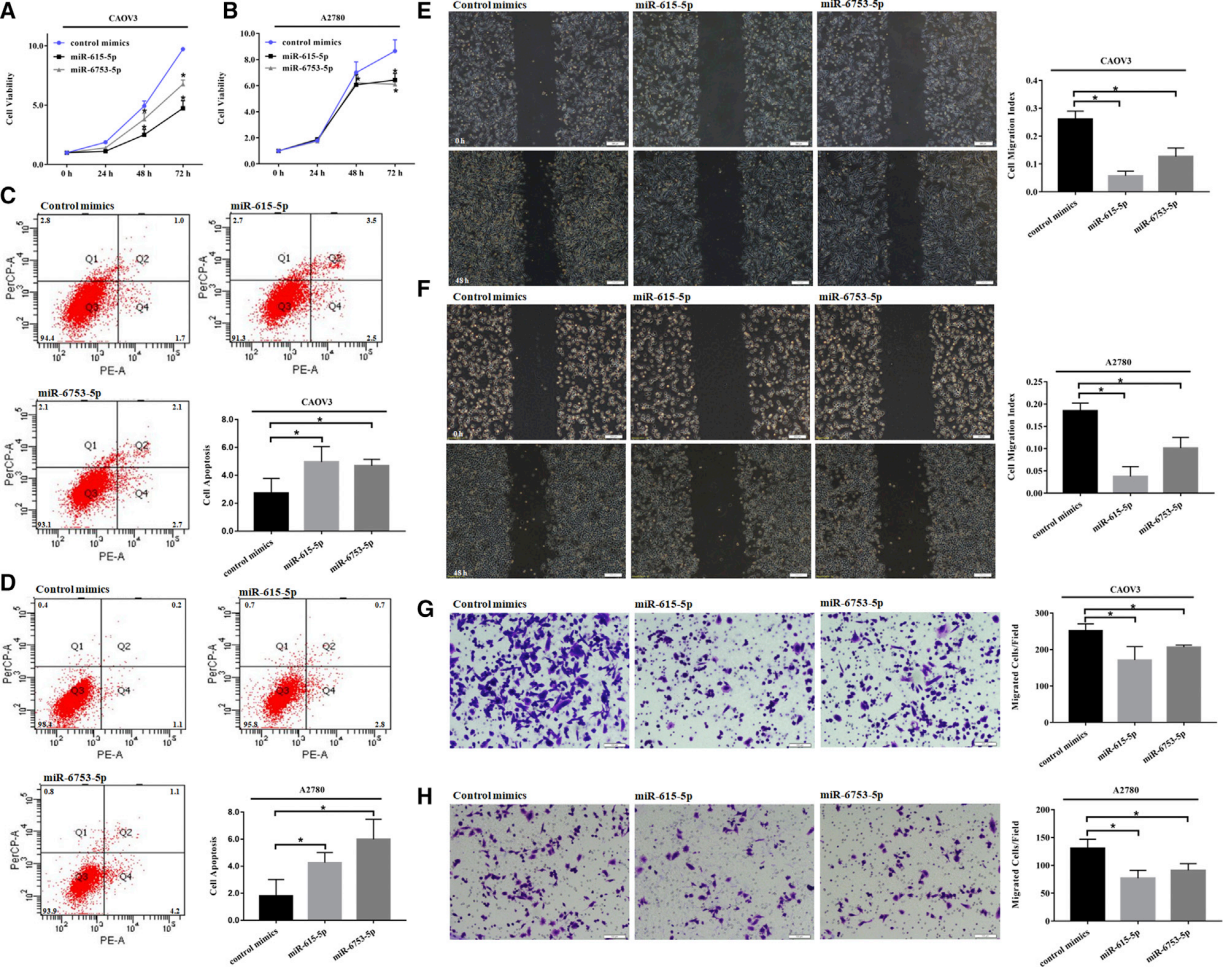
Effects of miR-615-5p and miR-6753-5p on Ovarian Cancer Cells (A, C, E, and G) Following transfection of CAOV3 cells with miR-615-5p or miR-6753-5p mimics, cell viability was suppressed (A), apoptosis was increased (C), and migration (E) and invasion ability (G) were inhibited. Similar results were found following transfection of A2780 cells with miR-615-5p or miR-6753-5p mimics (B, D, F, and H). The results shown are representative of three separate experiments; data are expressed as mean ± SD. *p < 0.05.

**Figure 4:**
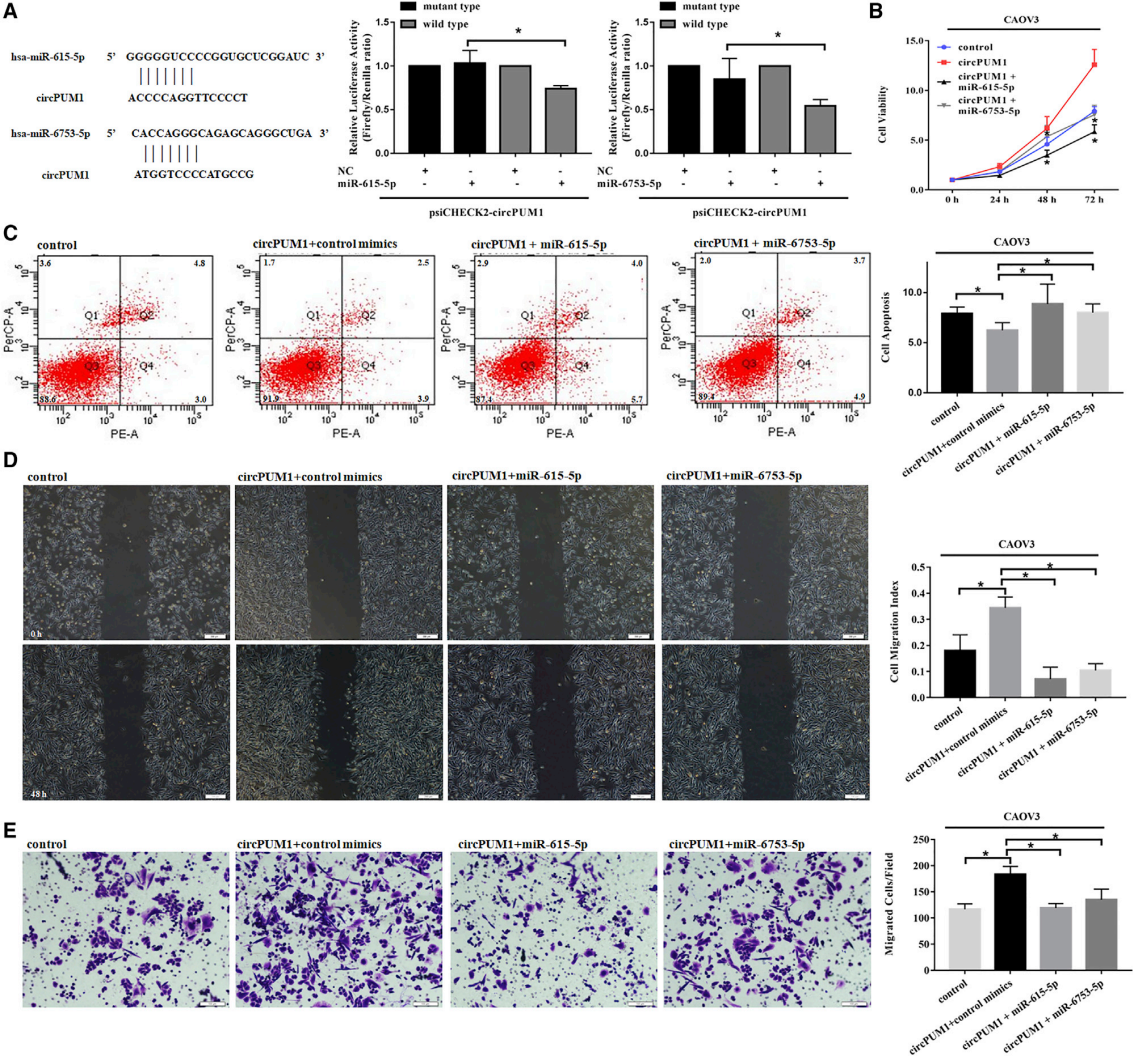
CircPUM1 Sponges miR-615-5p and miR-6753-5p Predicted complementary miR-615-5p and miR-6753-5p binding sequences around the circPUM1 splice sites (A). Dual-luciferase reporter assays showed that both miR-615-5p and miR-6753-5p significantly decreases the relative luciferase activity of the wild-type circPUM1 luciferase plasmid compared with the mutant (A). (B–E) Overexpression of miR-615-5p or miR-6753-5p reversed the effects of increased cell proliferation (B), migration (D), invasion (E), and decreased apoptosis (C) observed in circPUM1-overexpressing cells. Data represent the mean ± SD of three independent experiments. *p < 0.05.

Using a circRNA/miRNA interaction prediction website (http://www.mirdb.org/), we identified potential complementary miR-615-5p and miR-6753-5p binding sequences around the circPUM1 splice sites (Figures 3 and 4A). Dual-luciferase reporter assays indicated that both miR-615-5p and miR-6753-5p significantly decreased the relative luciferase activity of the wild-type circPUM1 luciferase plasmid compared with the mutant version (Figure 4A, p < 0.05).

### Effects of miR-615-5p and miR-6753-5p on Ovarian Cancer Cells

After transfection of CAOV3 and A2780 cells with miR-615-5p or miR-6753-5p, viability was suppressed (Figures 3A and 3B, p < 0.05), apoptosis increased (Figures 3C and 3D, p < 0.05), and cell migration (Figures 3E and 3F, p < 0.05) and invasion ability (Figures 3G and 3H, p < 0.05) were inhibited.

### Overexpression of miR-615-5p or miR-6753-5p Reverses the Effects of circPUM1

Overexpression of miR-615-5p or miR-6753-5p reversed the effects of increased cell proliferation (Figure 4B, p < 0.05), migration (Figure 4D, p < 0.05), invasion (Figure 4E, p < 0.05), and decreased apoptosis (Figure 4C, p < 0.05) in circPUM1 overexpressing cells.

### miR-615-5p and miR-6753-5p Regulate Expression of NF-κB and MMP2 Proteins, Respectively

**Figure 5:**
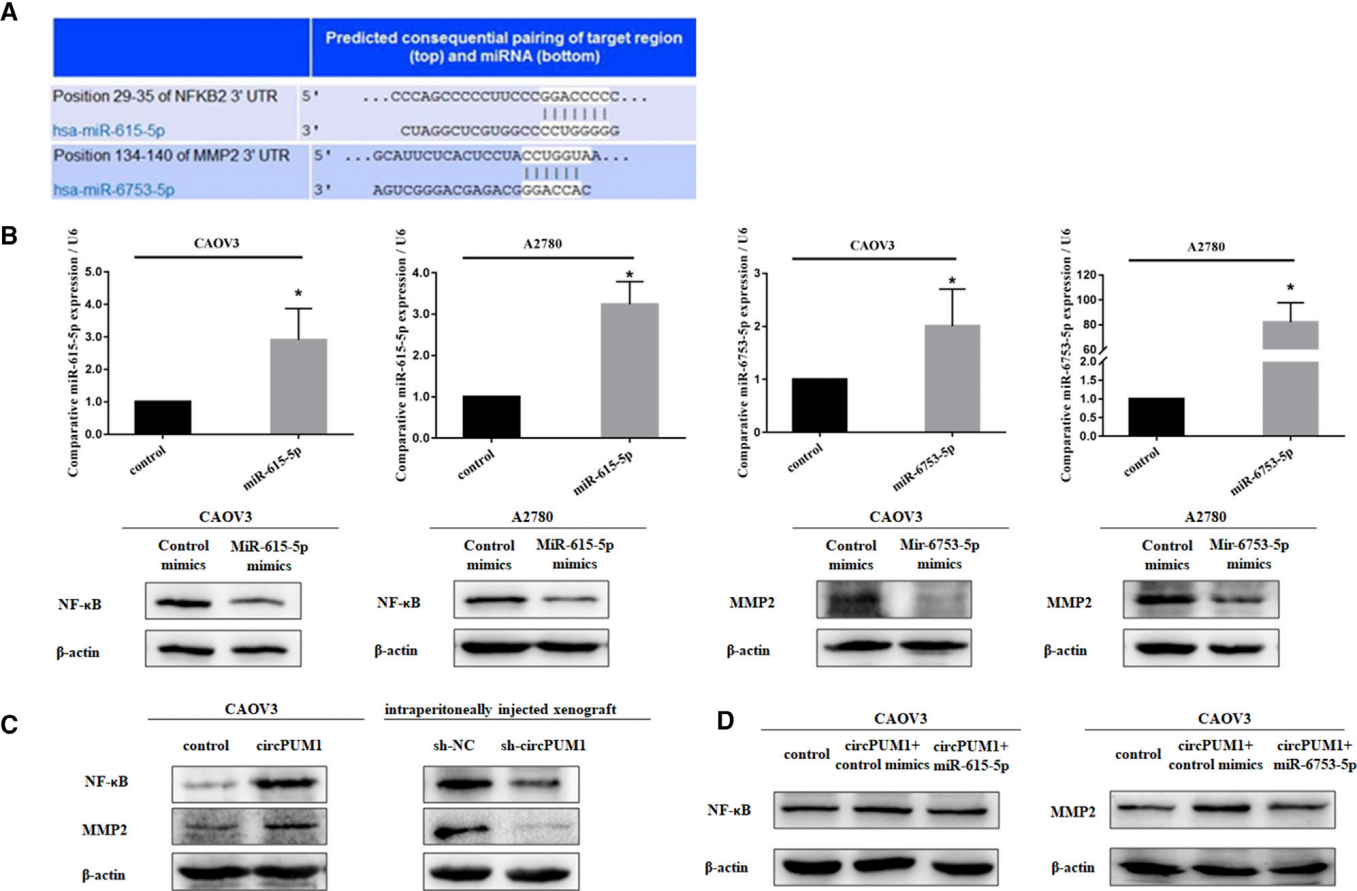
circPUM1 Regulates Protein Expression of NF-κB and MMP2 by Sponging miR-615-5p and miR-6753-5p Predicted complementary miR-615-5p and miR-6753-5p binding sequences in the 3′ UTR of NF-κB and MMP2 (A). NF-κB expression was downregulated after miR-615-5p transfection; MMP2 expression was downregulated after miR-6753-5p transfection (B). NF-κB and MMP2 expression was upregulated in circPUM1-overexpressing cells. Following i.p. injection of female BALB/c nude mice with circPUM1-knockdown A2780 cells, NF-κB and MMP2 expression levels were significantly lower than those in the control group (C). Overexpression of miR-615-5p or miR-6753-5p reversed the upregulation of NF-κB and MMP2 observed in circPUM1-overexpressing cells (D).

Using http://www.targetscan.org/, complementary miR-615-5p and miR-6753-5p binding sequences were predicted in the 3′ UTRs of NF-κB and matrix metallopeptidase 2 (MMP2), respectively (Figure 5A). Thus, we predicted that NF-κB and MMP2 are targets of miR-615-5p and miR-6753-5p, respectively.

Western blot analysis showed that compared with the control group, NF-κB and MMP2 expression were downregulated after transfection with miR-615-5p and miR-6753-5p, respectively (Figure 5B).

### circPUM1 Regulates Protein Expression of NF-κB and MMP2

Compared with the control group, circPUM1 overexpression upregulated the expression levels of NF-κB and MMP2. Silencing of circPUM1 yielded the opposite effects on i.p.-injected xenograft tumors (Figure 5C). Moreover, overexpression of miR-615-5p and miR-6753-5p reversed the upregulation of NF-κB and MMP2, respectively, in circPUM1-overexpressing cells (Figure 5D).

### Exosome Extraction and Identification

**Figure 6:**
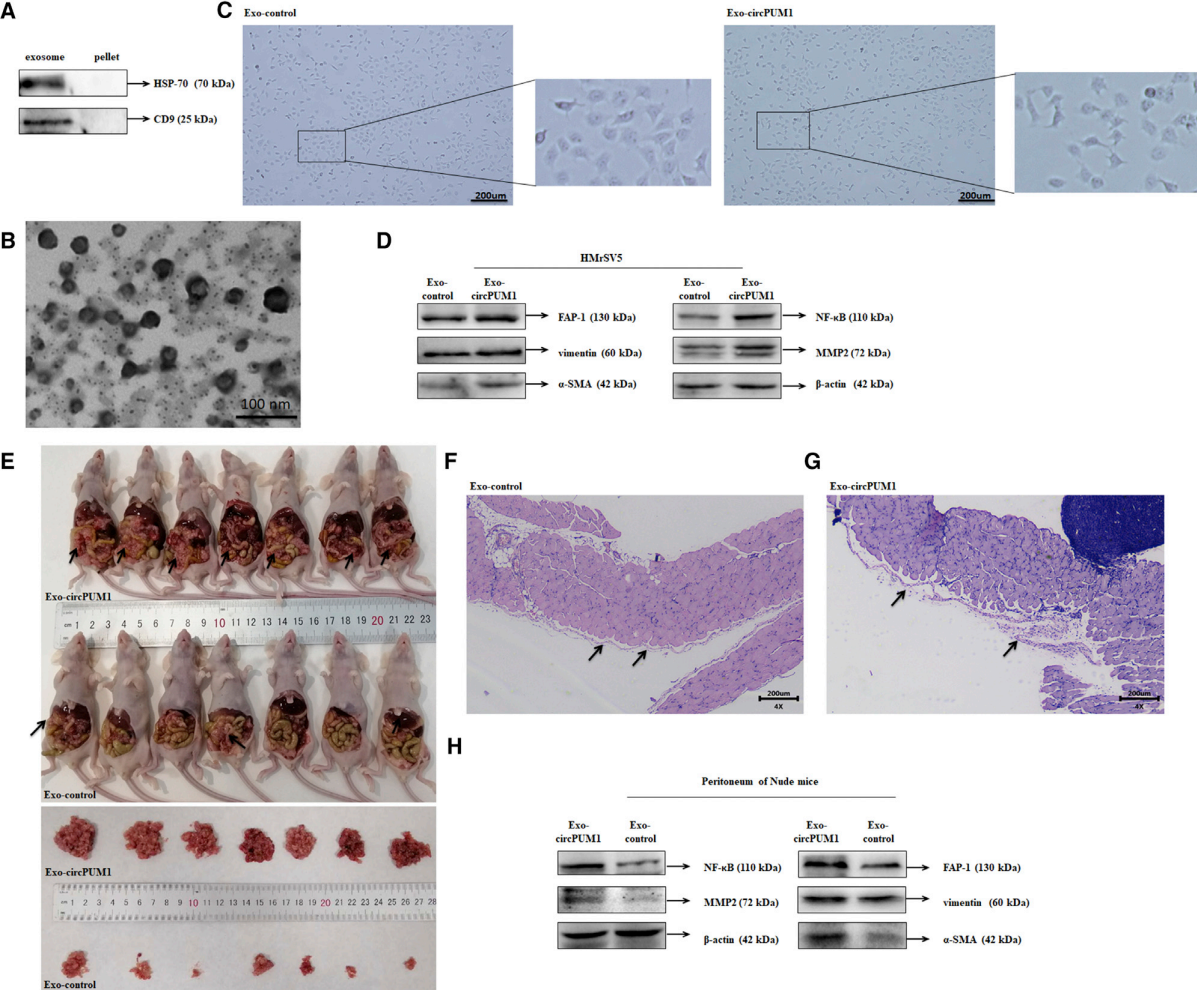
Exosomal circPUM1 Induces Mesothelial-to-Mesenchymal Transition and Promotes Tumor Peritoneal Dissemination Exosome-specific markers, such as CD9 and HSP70, were expressed in the exosome pellet (A). The diameters of the extracted exosomes ranged from 10 to 100 nm (B). After incubation with circPUM1 exosomes, HMrSV5 cells exhibited fibroblast-like cell morphology (C). Following i.p. injection of female BALB/c nude mice with CAOV3 cells, the number of tumor foci was significantly increased, and visible tumor nodules were disseminated throughout the peritoneum, mesentery, and liver in the group treated with circPUM1 exosomes compared with those treated with NC exosomes (E). H&E staining revealed that the peritoneum was covered with a layer of flat mesothelial cells with intact cellular junctions (F). In the circPUM1 exosome-treated group, mesothelial cells were arranged loosely, with some exhibiting spindle-shaped morphology with surrounding infiltration of neoplastic cells and reactive thickening of the stromal layer under the mesothelium (G). Expression of FAP-1, vimentin, α-SMA, NF-κB, and MMP2 was upregulated in the mesothelial cells and peritoneum of mice in the circPUM1 exosome-treated group (D and H).

Western blot analysis confirmed expression of exosome-specific markers, such as CD9 and HSP70, in the exosome pellet (Figure 6A). Electron microscopic evaluation of the extracted exosomes revealed a diameter of 10 to 100 nm (Figure 6B).

### Exosomal circPUM1 Regulates NF-κB and MMP2 Expression the Peritoneum Both *In Vitro* and *In Vivo*

qRT-PCR analysis confirmed higher circPUM1 expression in exosomes derived from circPUM1-overexpressing CAOV3 cells compared with the levels detected in CAOV3 cells (Table S3). Compared with the effects of incubation with exosomes from CAOV3 cells, HMrSV5 cells treated with exosomal circPUM1 were transformed into cells with fibroblast-like morphology (Figure 6C). To explore the effects exosomal circPUM1 on tumor peritoneal dissemination, we injected nude mice with exosomes after i.p. injection of CAOV3 cells. In the group treated with circPUM1 exosomes, the number of tumor foci was significantly increased and visible tumor nodules were disseminated throughout the peritoneum, mesentery, and liver (Figure 6E). H&E staining revealed that the peritoneum was covered with a layer of flat mesothelial cells. The mesothelial cells were arranged in a monolayer with intact cellular junctions (Figure 6F). In the circPUM1 exosome-treated group, mesothelial cells were arranged loosely, with some mesothelial exhibiting spindle-shaped morphology with surrounding infiltration of neoplastic cells and reactive thickening of the stromal layer under the mesothelium (Figure 6G). Western blot analysis showed that compared with the NC exosome group, expression of FAP-1, vimentin, α-SMA, NF-κB, and MMP2 was upregulated in circPUM1 exosome-treated HMrSV5 cells, as well as in the peritoneum (Figures 6D and 6H).

## Discussion

The relationship between the network of gene-expression regulation and its vital role in the development and progression of malignant tumors is gaining increasing attention.^16^ In particular, circRNA has become a new focus of research. Because of its stable structure and rich miRNA binding sites, circRNA can sponge various miRNAs and function as competing endogenous RNA.^17^^,^^18^ Previous studies have shown that circRNAs are involved in the molecular mechanism of malignancies such as bladder cancer,^19^ hepatocellular carcinoma,^20^ and breast cancer.^21^

In our previous study, we showed that the *PUM1* gene was closely related with tumorigenesis and progression in ovarian cancer. After interfering with the expression of PUM1 in the A2780 ovarian cancer cell line, cell proliferation, migration, and invasion ability were inhibited, while cell apoptosis increased.^15^ Thus, we hypothesized that its circular transcript has similar effects. In this study, we used qRT-PCR to quantify the expression levels of circPUM1 (circ_0000043; http://www.circbase.org/) in 62 cases of epithelial ovarian carcinoma and 13 cases of normal ovarian tissues. circPUM1 was significantly higher in ovarian carcinoma than in normal ovarian tissue and was positively related with International Federation of Gynecology and Obstetrics (FIGO) stage. These results indicate that circPUM1 correlates with the development of ovarian carcinoma.

To further explore the function of circPUM1 in ovarian cancer, we performed cell function assays both *in vitro* and *in vivo*. In the A2780 ovarian cancer cell line, the circular structure of circPUM1 was destroyed by shRNA targeting the splice sites, leading to suppression of cell proliferation, migration, and invasion and induction of cell apoptosis. In contrast, circPUM1 overexpression in the CAOV3 ovarian cancer cell line exerted the opposite effects. Furthermore, i.p. injection of circPUM1-knockdown tumor cells resulted in a decrease in the metastatic ability of the xenografted tumors in nude mice. These results indicate that circPUM1 plays a vital role in promoting tumorigenesis and progression of ovarian cancer.

In this study, we investigated the role of circPUM1 affect the development of ovarian cancer and the underlying molecular mechanism. Recently, accumulating evidence has confirmed that circRNAs can act as “miRNA sponges” to release the inhibitory effect of miRNA on target gene expression.^16^^,^^17^ miRNAs are approximately 22 nucleotides in length and characterized by specific binding with the 3′ UTR of target mRNAs.^22^ By blocking translation or inducing degradation of targets, miRNA participates in regulating gene expression at the post-transcriptional level. Abnormal expression of miRNAs is also closely related with tumor occurrence and development.^23^^,^^24^

Using bioinformatics prediction algorithms (http://www.mirdb.org/), we identified various potential complementary miRNA-binding sequences, including miR-615-5p and miR-6753-5p, around the circPUM1 splice sites. Fortunately, binding of these two miRNAs with circPUM1 was confirmed by dual-luciferase reporter assay. Furthermore, transfection of the ovarian carcinoma cell lines A2780 and CAOV3 with miR-615-5p and miR-6753-5p mimics reduced cell proliferation, migration, invasion, and increased cell apoptosis. In accordance with our results, miR-615-5p has been confirmed to play an inhibitory role in cell proliferation and migration in pancreatic ductal adenocarcinoma.^25^^,^^26^ Moreover, overexpression of miR-615-5p and miR-6753-5p reversed the increased cell proliferation, migration, invasion, and decreased apoptosis observed in cells overexpressing circPUM1.

We also predicted the targets of these two miRNAs (http://www.targetscan.org/). We found that miR-615-5p was able to bind to the 3′ UTR of the oncogene NF-κB, while miR-6753-5p was able to bind to the 3′ UTR of the oncogene MMP2. Western blot analysis showed that NF-κB expression was decreased in CAOV3 and A2780 cells transfected with miR-615-5p. Similarly, MMP2 was downregulated after miR-6753-5p transfection. These results revealed that NF-κB and MMP2 are targets of miR-615-5p and miR-6753-5p, respectively. Our results also showed that the expression levels of NF-κB and MMP2 were significantly decreased after knockdown of circPUM1. Overexpression of miR-615-5p or miR-6753-5p reversed the upregulation of NF-κB or MMP2 in cells overexpressing circPUM1, which suggests that circPUM1 mediates its carcinogenic effects by regulating the expression of these oncogenes in ovarian cancer.

The NF-κB transcription factor family has been reported to regulate a variety of important genes involved in carcinogenesis and progression, such as cell proliferation, angiogenesis, tumor invasion, and metastasis.^27^, ^28^, ^29^ Hernandez et al.^30^ reported that activation of NF-κB signaling increased the aggressiveness of ovarian cancer. Blockade of this signaling pathway significantly inhibited the expression of vascular endothelial growth factor (VEGF) and interleukin-8 (IL-8), two major proangiogenic molecules, and suppressed ovarian cancer cell implantation in the peritoneal cavity.^31^

As a member of zinc-dependent endopeptidase family, MMPs function as the main proteolytic enzyme in degradation of extracellular matrix (ECM), and are involved in tumor invasion, epithelial–mesenchymal transition (EMT), angiogenesis, and metastasis.^32^, ^33^, ^34^, ^35^ In ovarian cancer, MMP2 expression correlates with tumor stage grading and metastasis and is an independent risk prognostic factor in ovarian cancer.^36^, ^37^, ^38^ During the initial stages of ovarian cancer cell metastasis, MMP2 can destroy the protective barrier formed by intercellular tight junctions between mesothelial cells on the inner surface of the peritoneum and construct a favorable tumor microenvironment for peritoneal adhesion of ovarian cancer cell dissemination and peritoneal metastasis.^36^^,^^39^^,^^40^

Since we confirmed that circPUM1 promotes cell proliferation, migration, and invasion in ovarian cancer cells, we speculated that circPUM1 exists in cancer-secreted exosomes, from which it is transferred to act on distant organs, such as the peritoneum. Thus, we further investigated the ability of circPUM1, in the form of exosomes, to regulate the peritoneal expression of MMP2 and promote the dissemination of tumor foci and cancer progression.

To explore its underlying molecular mechanism, we extracted exosomes from both control and circPUM1-overexpressing CAOV3 cell supernatants by ultracentrifugation; these were verified by electron microscopy and western blot. PCR results showed high levels of circPUM1 in exosomes isolated from culture medium of circPUM1-overexpressing cells. In our study, after being cultured with exosomal circPUM1, HMrSV5 cells were transformed to exhibit a fibroblast-like morphology. Furthermore, the increased expression of FAP-1, vimentin, and α smooth muscle actin (α-SMA), indicated that exosomal circPUM1 induces mesothelial-to-mesenchymal transition (MMT).^41^^,^^42^ In addition, we found that i.p. injection of circPUM1 exosomes promoted cancer metastasis and peritoneal dissemination *in vivo*, with similar MMT changes found in peritoneum of xenografted nude mice. Moreover, we found increased expression of NF-κB and MMP2 in the peritoneum of xenografted nude mice treated with exosomal circPUM1 compared with the levels detected in mice treated with NC exosomes. Thus, we conclude that circPUM1 is secreted in exosomes and metastasizes to the peritoneum where it increases the expression of NF-κB and MMP2 in mesothelial cells and promotes peritoneal dissemination.

Taken together, our results show that circPUM1 upregulates NF-κB and MMP2 by sponging miR-615-5p and miR-6753-5p and releasing their inhibitory effect on the expression of these two targets, thus promotes tumorigenesis and progression of ovarian cancer. In addition, exosomal circPUM1 acts on peritoneal mesothelial cells to induce MMT, thus facilitating tumor metastasis. In conclusion, our investigation sheds light on the molecular mechanism by which circPUM1 not only promotes the proliferation, migration, and invasion of ovarian cancer cells, but also increases peritoneal spread of tumors in the form of cancer-derived exosomes.

## Materials and Methods

### Ovarian Cancer Specimens

Specimens were collected from 62 cases of epithelial ovarian carcinoma and 13 cases of normal ovarian tissues at the Department of Gynecology of the First Affiliated Hospital of China Medical University (Shenyang, China). None of the patients had undergone chemotherapy or radiotherapy prior to surgery. The samples were frozen in liquid nitrogen at −80°C for qRT-PCR. This study was approved by the China Medical University Ethics Committee. Informed consent was obtained from all the enrolled patients in accordance with ethical and legal standards.

### Cell Culture and Transfection

The human ovarian carcinoma cell line (A2780) was cultured in DMEM (HyClone, Logan, UT, USA). The CAOV3 and HMrSV5 were cultured in RPMI 1640 (HyClone) supplemented with 100 U/mL of penicillin/streptomycin and 10% fetal bovine serum (FBS). All cells were cultured at 37°C in an atmosphere containing 5% CO_2_. Cells were transfected using Lipofectamine 3000 (Invitrogen, Carlsbad, CA, USA) according to the manufacturer’s instructions.

To generate a circPUM1-overexpressing cell line, we stably transfected CAOV3 cells, which express low levels of circPUM1, with the pLCDH-circPUM1 plasmid (Geneseed Biotech, Guangzhou, China). To generate a circPUM1-knockdown cell line, we transfected A2780 cells, which express high levels of circPUM1, with sh-circPUM1-GFP (Hanbio Biotech, Shanghai, China) targeting the ring-forming sequences to break up its circular structure. After transfection, the circPUM1 overexpression and silenced cell lines were selected by culture in the presence of 2 μg/mL puromycin. The circPUM1 shRNA sequences were: 5′-GatccGAACAACAGGGCCCAAGGGATGCAGATTCAAGAGATCTGCATCCCTTGGGCCCTGTTGTTTTTTTTc-3′ (top strand) and 5′-aattgAAAAAAAACAACAGGGCCCAAGGGATGCAGATCTCTTGAATCTGCATCCCTTGGGCCCTGTTGTTCg-3′ (bottom strand). The circPUM1 sequence is shown in Table S3. The sequences of miR-615-5p mimics were: 5′-GGGGGUCCCCGGUGCUCGGAUC-3′ (sense), 5′-GAUCCGAGCACCGGGGACCCCC-3′ (antisense). The sequences of miR-6753-5p mimics were: 5′-CACCAGGGCAGAGCAGGGCUGA-3′ (sense), 5′-UCAGCCCUGCUCUGCCCUGGUG-3′ (antisense).

### MTT Assay

Cell proliferation was evaluated by MTT assay. Cells were seeded in 96-well plates at a density of 3,000 cells per well. At 0 h, 24 h, 48 h, and 72 h after seeding, 20 μL of 5 mg/mL tetrazolium (MTT) solution (Solarbio, Beijing, China) was added to the cells. After incubation for 3 h at 37°C, the supernatant was removed and 150 μL of DMSO was added to dissolve the precipitated formazan. The absorbance at 490 nm was detected with a microplate spectrophotometer after shaking.

### Cell Apoptosis Assay

After release from 6-well plates, the cells were washed twice with PBS. The cells were collected by centrifugation at 1,500 rpm for 5 min and then stained with 5 μL of 7AAD and PE-labeled Annexin V (BD Biosciences) in 500 μL of 1 × Binding Buffer. The cell apoptosis rate was determined by a flow cytometry within 1 h.

### Wound-Healing Assay

When the cells were cultured to 80% confluence, the wounds were introduced in the form of a scratch using a 200 μL pipette tip. Subsequently, the cells were washed with PBS and then cultured in FBS-free medium containing 20 μg/mL of mitomycin C to suppress cell proliferation. The wounds were photographed at 0 h and 48 h after scratching and the wound-healing rate was calculated as a reflection of cell migration ability.

### Cell Invasion Assay

Cell invasion was detected using the Matrigel-coated Transwell method. The filters were coated with 30 μL Matrigel and 50,000 cells in 200 μL of serum-free medium were seeded in the upper compartment of the Transwell chambers, while 600 μL of medium supplemented with 10% FBS was added to the lower compartment. After 48 h of incubation at 37°C, cells above the upper surface of the filter were removed, while the cells on the bottom of the filter were fixed with formaldehyde and then stained with crystal violet.

### qRT-PCR

Total RNA was extracted from the ovarian carcinoma cell lines and tissues using TRIzol reagent. After measuring OD_260/280_ with a spectrophotometer (Unico, Shanghai, China), 5 μg of quantified RNA was reverse-transcribed to complementary DNA with transcriptase using random primers (TaKaRa). SYBR Premix Ex Taq II kit (TaKaRa, Shiga, Japan) was used to amplify the cDNA. Comparative expression of the target genes was determined using 18S rRNA (18 S) as a reference gene with the 2^-ΔΔCt^ method. Mature miR-615-5p and miR-6753-5p were quantified by using hairpin-it miRNA qPCR quantitation kit (GenePharma, China) with U6 normalization.

### Western Blot Analysis

Total protein was extracted with radioimmunoprecipitation assay (RIPA) lysis buffer. The cell lysate was then quantified and boiled for 5 min before 30 μg of the samples were separated by10% SDS-PAGE. Proteins were then electrotransferred to polyvinylidene fluoride (PVDF) membranes (Millipore, USA) and blocked for 2 h with 3% BSA at room temperature. Subsequently, the membranes were incubated overnight at 4°C with primary antibodies for the detection of NF-κB2 (1:1,000; Proteintech, USA), MMP2 (1:1,000; Proteintech), HSP70 (1:500; Boster, USA), CD9 (1:1,000; Proteintech), FAP-1 (1:1,000; Abbkine, USA), vimentin (1:1,000; Proteintech), α-SMA (1:1,000; Proteintech), and β-actin (1:3,000; Proteintech). The membranes were then washed (3 × 10 min) with Tris-buffered saline containing Tween-20 (TBST). After incubation for 90 min at room temperature with anti-rabbit secondary detection antibodies (1:5,000; Proteintech), immunoreactive bands were visualized by enhanced chemiluminescence (Santa Cruz Biotechnology, Santa Cruz, CA, USA).

### *In Vivo* Xenograft Tumor Model

Female BALB/c nude mice (aged 5 weeks; Vital River Laboratories, Beijing, China) were i.p. injected with 1 × 10^7^ circPUM1-knockdown A2780 cells (or mock transfected cells) suspended in serum-free medium (6 mice per group). The length (L) and width (W) of the tumor were measured with a caliper, and tumor volume was determined as tumor volume (mm^3^) = L × W^2^/2. The mice were sacrificed after 4 weeks and i.p. metastasis tumor lesions were excised, measured, and photographed.

To investigate the function of circPUM1 exosomes, we i.p. injected BALB/c nude mice with 1 × 10^7^ CAOV3 cells. After 3 days, the mice were divided into two groups (7 mice per group). One group received 200 μg of exosomes secreted from circPUM1-overexpressing CAOV3 cells, and the other group were injected with exosomes from control CAOV3 cells as a negative control (NC). The mice received a total of six injections in delivered every two days, and were sacrificed after 3 weeks. All the mice were maintained in a specific pathogen-free environment at the Experimental Animal Center of China Medical University.

### Extraction of Exosomes

Based on exosome purification protocols, cells were cultured in Exo-Clear cell growth medium (SBI, USA) for 36 h before collection. The supernatant was filtered using a 0.22 μm Millipore Express PES Membrane (Millex, USA) to remove dead cells. The filtrate was then centrifuged at 10,000 × *g* for 30 min to eliminate cell debris (Optima XPN-100 Ultracentrifuge, Beckman, USA). After ultracentrifugation at 100,000 × *g* for 70 min, the pellet containing exosomes was washed in PBS and then centrifuged at 100,000 × *g* for 70 min to remove contaminant proteins. Exosomes in the co-culture system were enriched using the Hieff Quick exosome isolation kit (for Cell Culture Media) (Yeasen, China) according to the instructions.

### Transmission Electron Microscopy

The exosome pellet was diluted with PBS and 5 μL was dripped onto a carbon-coated copper grid. After drying at 37°C for 15 min, the grid was stained with 20 g/L phosphotungstic acid for 3 min at room temperature. The exosomes were then observed using a transmission electron microscope (H-7650).

### Cell Morphology Evaluation

HMrSV5 cells were starved by cultivation in serum-free medium for 12 h before co-culture with circPUM1 exosomes isolated from control or circPUM1-overexpressing cell conditioned medium for 24 h. The morphology of cells was photographed under a light microscope.

### H&E Staining

The peritoneum of nude mice was excised and fixed in paraformaldehyde. After conventional dehydration and paraffin-embedding, sections were prepared, dewaxed with xylene, and dehydrated with a gradient series of alcohols. After washing with distilled water for 2 min, the sections were stained with hematoxylin for 4 min followed by eosin for 1 min. Sections were dehydrated and transparent, sealed, and observed under an Nikon light microscope (×4 magnification).

### Dual-Luciferase Reporter Assay

Dual-luciferase reporter plasmids were generated by cloning the miR-615-5p and miR-6753-5p binding sequences of circPUM1 into PSI-check2 dual-luciferase vectors (Hanbio Biotechnology, Shanghai, China). HEK293T cells were co-transfected with wild-type or mutated dual-luciferase plasmid, as well as miR-615-5p or miR-6753-5p mimics and scrambled mimics as controls. After induction for 48 h, luciferase activity was detected with the Dual-Luciferase Reporter Assay System (Promega, Madison, WI, USA). The relative luciferase signal was presented as firefly luciferase activity normalized to renilla luciferase activity.

### Statistical Analysis

All the data were presented as the mean ± SD of at least three independent experiments. The data were analyzed with SPSS 18.0 software using a two-tailed Student’s t test. p < 0.05 was considered to indicate statistical significance.

## Author Contributions

Y.Z. designed the research; X.G. performed the experiments and wrote the paper; Z.-h.Z. conducted the experiments; Y.L., S.C., and L.-l.W. performed the experiments and analyzed the data. All authors read and approved the final manuscript.

## Conflicts of Interest

All authors declare no competing interests.

## Supplemental Information

Document S1. Tables S1–S3

Document S2. Article plus Supplemental Information
