# Supplementary material for: circPUM1 Promotes Tumorigenesis and Progression of Ovarian Cancer by Sponging miR-615-5p and miR-6753-5p
Source: Mol Ther Nucleic Acids. 2019 Oct 23;18:882–92. doi: 10.1016/j.omtn.2019.09.032 (PMC6881671; doi:10.1016/j.omtn.2019.09.032)
Supplement: Document S1. Tables S1–S3 [file mmc1.pdf]

OMTN, Volume *18*

## Supplemental Information

# circPUM1 Promotes Tumorigenesis and Progression of Ovarian Cancer by Sponging miR-615-5p and miR-6753-5p

**Xue Guan, Zhi-hong Zong, Yao Liu, Shuo Chen, Li-li Wang, and Yang Zhao**

**Supplementary Table 1:** circPUM1 expression in normal ovary and ovarian carcinoma tissues

| **Groups** | **N** | **circPUM1 expression / 18s** | ***P* value** |
|---|---|---|---|
| Normal ovary | 13 | 4.37E-07 ± 5.17E-07 | ***0.0001*** |
| Ovarian carcinoma | 62 | 4.30E-06 ± 9.39E-06 | |

Bold and Italics means P < 0.05.

**Supplementary Table 2:** Correlation of circPUM1 expression with different clinicopathological features of ovarian carcinoma

| **Clinicopathological features** | **N** | **circPUM1 expression / 18s** | ***P* value** |
|---|---|---|---|
| **The pathology types** | | | 0.438 |
| Serous carcinoma | 54 | 4.26E-06 ± 9.94E-06 | |
| The other pathology types | 8 | 4.58E-06 ± 4.44E-06 | |
| **Age** | | | 0.432 |
| ≤ 52 | 33 | 4.11E-06 ± 1.13E-05 | |
| > 52 | 29 | 4.51E-06 ± 6.87E-06 | |
| **FIGO stages** | | | ***0.031*** |
| I-II | 19 | 1.91E-06 ± 3.04E-06 | |
| III-IV | 43 | 5.36E-06 ± 1.10E-05 | |
| **Pathology classification** | | | 0.122 |
| Well | 10 | 2.60E-06 ± 3.11E-06 | |
| Mod + Poor | 52 | 4.63E-06 ± 1.02E-05 | |

Bold and Italics means P < 0.05.

**Supplementary Table 3:** The expression level of circPUM1 in exosomes derived from NC and circPUM1-overexpressing CAOV3 cells.

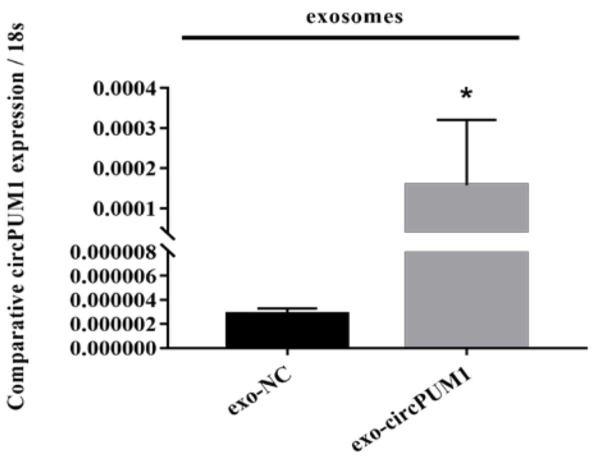


The DNA sequence and primer sequences of circPUM1.

>hsa_circ_0000043|NM_014676|PUM1|438bp

GGCCCAAGGGATGCAGACAGTGATGAAAACGACAAAGGTGAAAAGAAGA
ACAAGGGTACGTTTGATGGAGATAAGCTAGGAGATTTGAAGGAGGAGGGT
GATGTGATGGACAAGACCAATGGTTTACCAGTGCAGAATGGGATTGATGCA
GACGTCAAAGATTTTAGCCGTACCCCTGGTAATTGCCAGAACTCTGCTAAT
GAAGTGGATCTTCTGGGTCCAAACCAGAATGGTTCTGAGGGCTTAGCCCAG
CTGACCAGCACCAATGGTGCCAAGCCTGTGGAGGATTTCTCCAACATGGA
GTCCCAGAGTGTCCCCTTGGACCCCATGGAACATGTGGGCATGGAGCCTCT
TCAGTTTGATTATTCAGGCACGCAGGTACCTGTGGACTCAGCAGCAGCAAC
TGTGGGACTTTTTGACTACAATTCTCAACAACAG

hsa_circ_0000043（Divergent primers）

hsa_circ_0000043-F2: 5'- gcatggagcctcttcagtttg -3'

hsa_circ_0000043-R1: 5'- ccattggtcttgtccatcac -3'
